# Antifibrotic treatment improves clinical outcomes in patients with idiopathic pulmonary fibrosis: a propensity score matching analysis

**DOI:** 10.1038/s41598-020-72607-1

**Published:** 2020-09-24

**Authors:** Jieun Kang, Minkyu Han, Jin Woo Song

**Affiliations:** 1 Department of Pulmonary and Critical Care Medicine, Asan Medical Center, University of Ulsan College of Medicine, 88 Olympic-Ro 43-gil, Songpa-gu, Seoul, 05505 Republic of Korea; 2 Department of Clinical Epidemiology and Biostatistics, Asan Medical Center, Seoul, Republic of Korea

**Keywords:** Diseases, Respiratory tract diseases, Health care, Therapeutics, Drug therapy

## Abstract

In patients with idiopathic pulmonary fibrosis (IPF), the effects of antifibrotic agents on the prognosis remain unclear. This study aimed to investigate the impact of antifibrotic treatment on the risks of mortality, hospitalisation, and acute exacerbation in real-world patients with IPF. A total of 1213 IPF patients (biopsy-proven cases: 405) were included in this retrospective study. Propensity score matching was used to adjust for differences in baseline characteristics between patients who received antifibrotic treatment and who did not. A Cox proportional hazard model was used to compare the risks of all-cause mortality, hospitalisation, acute exacerbation, and mortality following acute exacerbation between the two groups. From the 1213 patients, 474 matched pairs were generated. The mean age of the patients in the matched cohort was 65.8 years and 82.8% were men. The median follow-up duration was 27 months. Antifibrotic treatment significantly reduced the risks of mortality [hazard ratio (HR), 0.59; 95% confidence interval (CI), 0.48–0.72; p < 0.001], all-cause hospitalisation (HR 0.71), respiratory-related hospitalisation (HR 0.67), acute exacerbation (HR 0.69), and mortality after acute exacerbation (HR 0.60). Our results suggest that antifibrotic treatment may reduce the risks of all-cause mortality, hospitalisation, acute exacerbation, and mortality after acute exacerbation in patients with IPF.

## Introduction

Idiopathic pulmonary fibrosis (IPF) is a progressive fibrosing interstitial lung disease with a poor prognosis^1^. Most patients with IPF experience frequent hospitalisations for respiratory and/or non-respiratory causes during the course of the disease^2–4^. As a result, this condition is associated with a high economic burden^3^ and short-term mortality^5^. Notably, acute exacerbation (AE) results in in-hospital deaths in approximately 50% of the cases, and seriously impacts the prognosis of IPF patients^6^. Therefore, assessing how a drug affects the risks of hospitalisation, and AE, in addition to mortality is important when evaluating the efficacy of treatments in patients with IPF.

Both pirfenidone and nintedanib were shown to significantly reduce the rate of decline in forced vital capacity (FVC) in IPF patients in previous clinical trials^7–9^. Since this discovery, several pooled analyses have been conducted to evaluate the effect of antifibrotic agents on clinical outcomes aside from FVC. Pirfenidone was associated with a significant reduction in the risks of mortality^10^, respiratory-related hospitalisation, and death after hospitalisation^11^, whereas nintedanib was associated with lower risks of on-treatment mortality and AE rate^12^. However, these results bear a fundamental limitation in that they were based on clinical trial data; the patients evaluated were a pharmaceutical cohort selected according to strict inclusion criteria and may not represent real-world patients with various comorbidities and different levels of disease severity.

So far, observational studies have evaluated the effect of antifibrotic treatment in a real-world setting^13–19^. Correlating with the findings of the previously mentioned clinical trials, these studies showed that antifibrotic treatment reduced FVC decline rate^13–16^ and mortality^17–19^, but most of the studies included a small number of patients and were underpowered. Recently, Dempsey et al. reported a mortality benefit of antifibrotic treatment in a large cohort of IPF patients based on an insurance database. However, the study included mostly white patients and Asian population comprised only 3.0% of the total subject. More importantly, evidence demonstrating that antifibrotic treatment reduces the risk of AE was not present. In this study, we aimed to analyse the impact of antifibrotic agents on the risks of all-cause mortality, hospitalisation, and AE in IPF patients in clinical practice.

## Methods

### Study subjects

**Figure 1:**
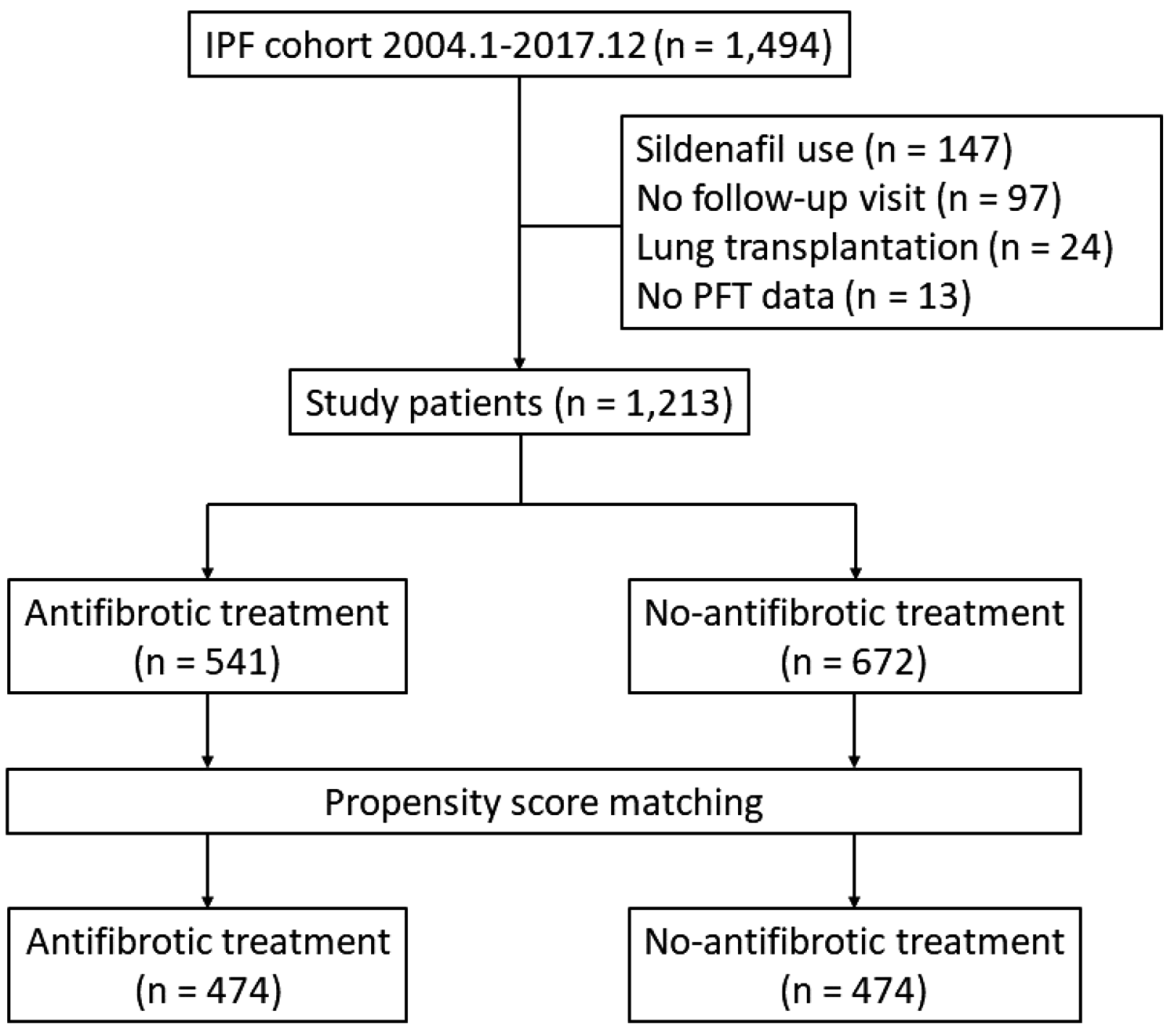
Flowchart of patient selection. *IPF* idiopathic pulmonary fibrosis, *PFT* pulmonary function test.

Between January 2004 and December 2017, a total of 1494 patients with IPF were diagnosed at Asan Medical Center, Seoul, Republic of Korea. Patients were excluded if they (1) received concomitant sildenafil, (2) did not attend a follow-up visit after diagnosis, (3) underwent lung transplantation, or (4) did not have baseline pulmonary function test results. Finally, 1213 IPF patients (biopsy performed in 405 patients) were included in this study (Fig. 1). Some of these patients had been included in previous studies^6,20–22^. All patients fulfilled the IPF diagnostic criteria of the American Thoracic Society (ATS), European Respiratory Society (ERS), Japanese Respiratory Society, and Latin American Thoracic Association^1^. The diagnosis of each patient was made through a multidisciplinary discussion.

To assess the effect of antifibrotic agents, patients were classified into two groups: an antifibrotic group and a no-antifibrotic group. The antifibrotic group comprised patients who received either pirfenidone or nintedanib for the treatment of IPF at least once and the no-antifibrotic group comprised patients who did not receive antifibrotic agents during the study period. The index date was set as the date of the first prescription of an antifibrotic agent in the antifibrotic group and the date of IPF diagnosis in the no-antifibrotic group. Patients were followed from the index date until the occurrence of the study outcome or June 30, 2019. This study was approved by the Institutional Review Board of Asan Medical Center (No.: 2019-0735), and the requirement for informed consent was waived due to the retrospective nature of the study. All methods were performed in accordance with the relevant guidelines and regulations of the journal.

### Study data and outcomes

Clinical and survival data for all patients were retrospectively obtained from medical records, telephone interviews, and/or the records of the National Health Insurance of Korea. Spirometric parameters^23^, diffusing capacity of the lung for carbon monoxide (DL_CO_)^24,25^, and total lung capacity^26^ were measured according to the ERS/ATS recommendations, and the results were presented as percentages of the normal predicted values.

The study outcomes included the risks of all-cause mortality, all-cause hospitalisation, respiratory- and non-respiratory-related hospitalisation, AE, and mortality after AE. Respiratory-related hospitalisation was defined as an unexpected admission due to acute respiratory worsening such as pneumonia, pneumothorax, pulmonary embolism, and AE. Non-respiratory-related hospitalisation was defined as an unexpected admission because of a non-respiratory problem such as acute coronary syndrome. AE was defined according to the criteria suggested by Collard et al.^27^ Follow-up visit usually every 3–6 months and hospitalisation records were reviewed to identify the development of the study outcomes.

### Statistical analysis

All values are expressed as mean ± standard deviation for continuous variables or as percentages for categorical variables. The student’s t-test was used for continuous data, and Pearson’s chi-squared test or Fisher’s exact test was used for categorical data.

We performed propensity score matching to adjust for differences in baseline characteristics between the antifibrotic and no-antifibrotic groups. The matched variables were age, sex, body mass index (BMI), FVC, DL_CO_, corticosteroid use in the 6 months prior to the index date. Survival was evaluated by Kaplan–Meier survival analysis and the log rank test. The relative risks of mortality, hospitalisation, and AE were analysed using a Cox proportional hazards model.

We also performed additional analyses in study subjects that did not exclude those treated with sildenafil. Sildenafil treatment and Charlson Comorbidity Index were matched in addition to the aforementioned variables. Study outcomes were investigated in these patients to see whether the inclusion of sildenafil users alter the study results. All p-values were two-tailed, and statistical significance was set at p < 0.05. All statistical analyses were performed using SPSS software (version 22.0; IBM Corporation, Somers, NY, USA) or R version 3.3.3 (R Foundation for Statistical Computing, Vienna, Austria).

## Results

### Baseline characteristics

The median follow-up duration of all 1213 patients was 27 months (interquartile range: 17–44 months). The mean age of the patients was 66.4 years and 82.0% were men. Antifibrotic treatment was administered to 541 patients (44.6%) (Fig. 1). In the unmatched cohort, patients without antifibrotic treatment were older and had a lower BMI, FVC, and DL_CO_ than those who received antifibrotic treatment (Table 1). Propensity score matching was performed to adjust for these differences, and 474 matched pairs were created. The baseline characteristics of the patients included in the matched cohort and those excluded are shown in Supplementary Table S1. The patients excluded from the matched cohort were older and showed greater lung function than those included.

**Table 1:** Comparison of baseline characteristics between the antifibrotic and no-antifibrotic groups of patients with idiopathic pulmonary fibrosis.

	Unmatched groups		p-value	Matched groups		p-value
	Antifibrotic	No-antifibrotic		Antifibrotic	No-antifibrotic	
No. of patients	541	672		474	474	
Age, years	65.5 ± 7.8	67.2 ± 8.1	< 0.001	65.8 ± 7.8	65.8 ± 8.3	0.904
Male	451 (83.4)	544 (81.0)	0.293	392 (82.7)	393 (82.9)	> 0.999
BMI	25.0 ± 3.0	23.8 ± 3.1	< 0.001	24.8 ± 2.8	24.5 ± 3.0	0.212
Smoking status			0.929			0.500
Current	68 (12.6)	80 (11.9)		63 (13.3)	55 (11.6)	
Former	344 (63.6)	433 (64.4)		298 (62.9)	315 (66.5)	
Never	129 (23.8)	159 (23.7)		113 (23.8)	104 (21.9)	
FVC	66.2 ± 13.7	70.6 ± 17.8	< 0.001	67.1 ± 13.4	67.0 ± 16.7	0.923
DL_CO_	52.9 ± 15.2	57.2 ± 19.3	< 0.001	53.3 ± 15.2	53.3 ± 18.0	0.963
Charlson comorbidity index	1.8 ± 1.0	1.9 ± 1.1	0.469	1.8 ± 1.0	1.9 ± 1.1	0.762

Data are presented as mean ± standard deviation or number (%) unless otherwise indicated.

*BMI* body mass index, *DL*_*CO*_ diffusing capacity of the lung for carbon monoxide, *FVC* forced vital capacity, *No.* number.

The mean age of the total patients in the matched cohort was 65.8 years and 82.8% were men (Supplementary Table S1). The median follow-up duration was 27 months (antifibrotic: 24 months vs. no-antifibrotic: 33 months, p < 0.001) and the median time from diagnosis to start of antifibrotic treatment was 11 months (interquartile range, 1–43 months). The median duration of antifibrotic treatment was 16 months. In the antifibrotic group, pirfenidone was the most commonly used medication and was prescribed to 429 patients (90.5%). Nintedanib was administered to 85 patients (17.9%). Forty patients (8.4%) were administered pirfenidone and nintedanib in sequence; treatment was switched from pirfenidone to nintedanib (6.8%) or vice versa (1.7%) due to intolerance. High-dose N-acetylcysteine (NAC) was prescribed to 290 patients (61.2%) in the no-antifibrotic group, whereas 1 patient (0.2%) in the antifibrotic group received high-dose NAC alongside pirfenidone.

### Clinical course and survival

**Figure 2:**
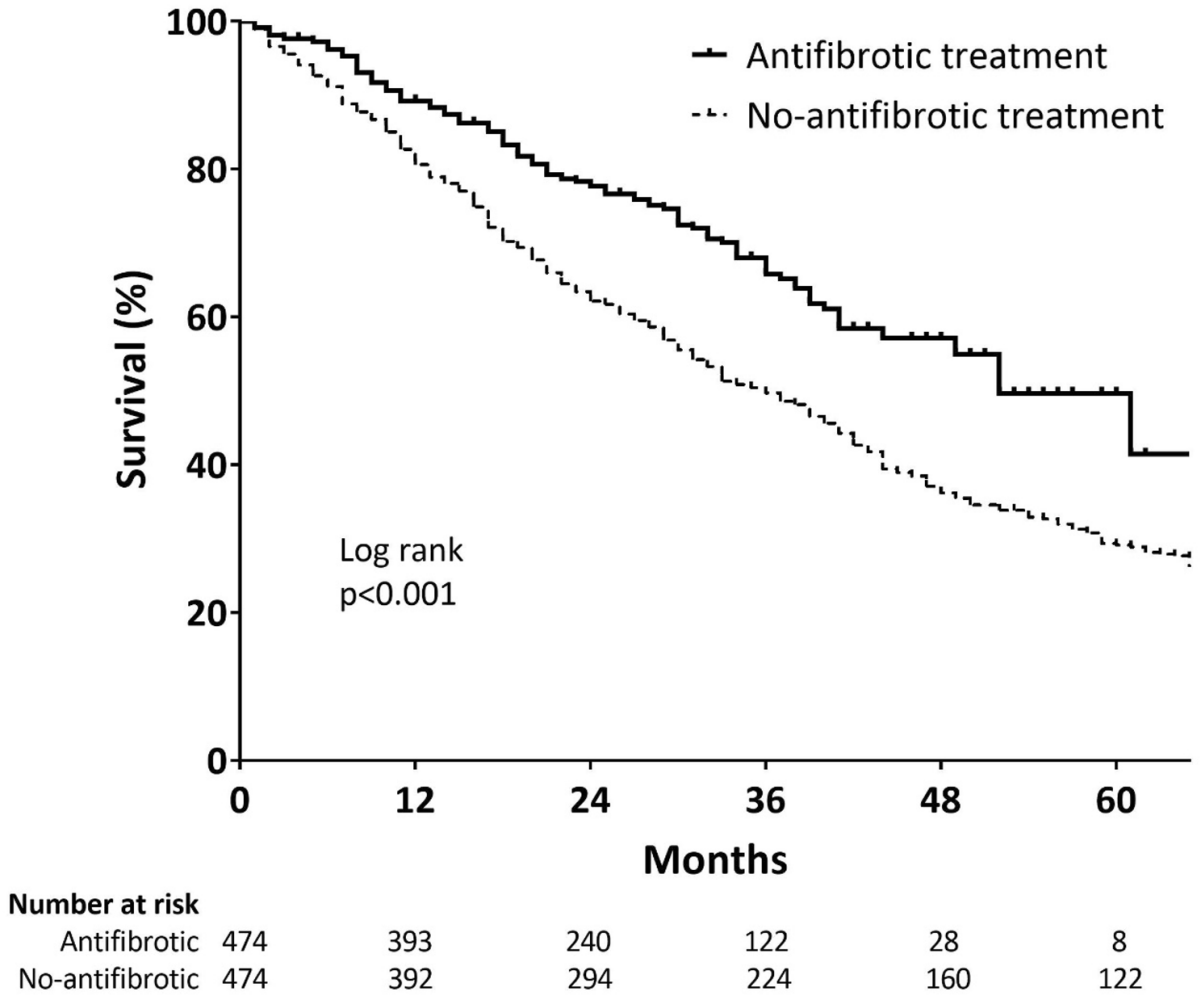
Comparison of survival curves between the antifibrotic and no-antifibrotic groups of patients with idiopathic pulmonary fibrosis. Kaplan–Meier survival curves are shown for the antifibrotic (solid line) and no-antifibrotic (dotted line) groups.

Table 2 shows the number of patients in the matched cohort who experienced at least one study outcome. A total of 522 patients (55.1%) died during the study period, and the median survival duration was 42 months (95% confidence interval [CI], 39.0–45.0). The antifibrotic group exhibited a significantly longer survival duration (median: 52 vs. 36 months; p < 0.001) than the no-antifibrotic group (Fig. 2). The annual mortality rates were also significantly lower in the antifibrotic group (1-year: 10.7% vs. 19.4%; 3-year: 34.2% vs. 50.3%; 5-year: 50.3% vs. 70.9%, all p < 0.001).

**Table 2:** Comparison of clinical outcomes between the antifibrotic and no-antifibrotic groups.

	Antifibrotic	No-antifibrotic	p-value
Number of patients	474	474	
All-cause mortality	137 (28.9)	385 (81.2)	< 0.001
Hospitalisation			
All-cause	141 (29.7)	221 (46.6)	< 0.001
Respiratory-related	105 (22.2)	175 (36.9)	< 0.001
Non-respiratory-related	42 (8.9)	77 (16.2)	0.001
Acute exacerbation	61 (12.9)	104 (21.9)	< 0.001
Mortality after acute exacerbation	45 (73.8)	104 (100.0)	< 0.001

Data are presented as number (%).

**Figure 3:**
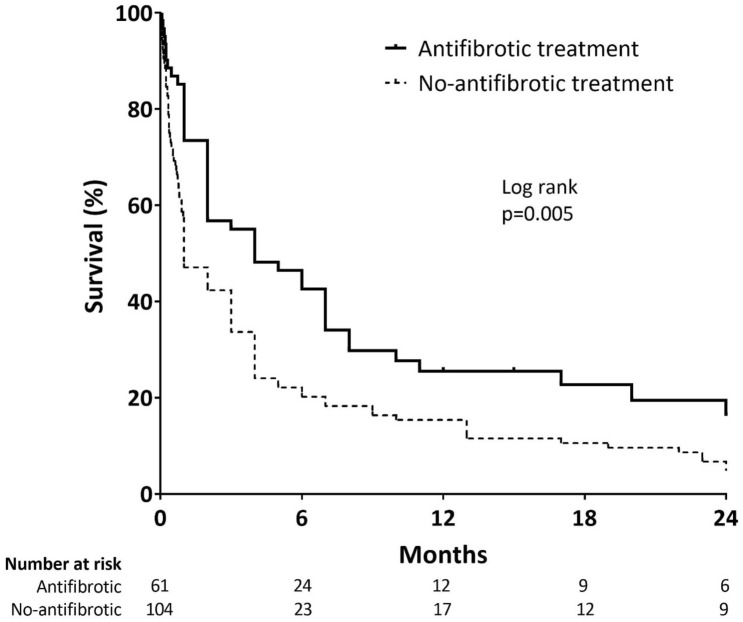
Comparison of survival after the development of acute exacerbation between the antifibrotic and no-antifibrotic groups. Kaplan–Meier survival curves are shown for the antifibrotic (solid line) and no-antifibrotic (dotted line) groups.

A total of 362 patients (38.2%) experienced unexpected hospitalisation at least once during follow-up. Overall, 243 patients (25.6%) experienced respiratory-related hospitalisations, 82 (8.6%) experienced non-respiratory-related hospitalisations, and 37 (3.9%) experienced both. Furthermore, 195 patients (20.6%) experienced AE, and 149 of them (90.3%) died after the development of AE during follow-up. As shown in Table 2, significantly fewer patients experienced hospitalisation (respiratory- and non-respiratory- related) and AE in the antifibrotic group. In addition, the number of deaths after the development of AE was significantly lower in the antifibrotic group. The median survival duration after AE was 4 months in the antifibrotic group and 1 month in the no-antifibrotic group (p < 0.001, Fig. 3).

The causes of the first respiratory-related hospitalisation of the patients (n = 280) in the matched cohort are shown in Supplementary Table S2. In both the antifibrotic and no-antifibrotic groups, the most common cause was AE (44.0% and 43.8%) followed by focal pneumonia (32.0% and 37.1%). There was no statistically significant difference in the cause of respiratory-related hospitalisation between the antifibrotic and no-antifibrotic groups (p = 0.381).

### Impact of antifibrotic treatment on prognosis

**Figure 4:**
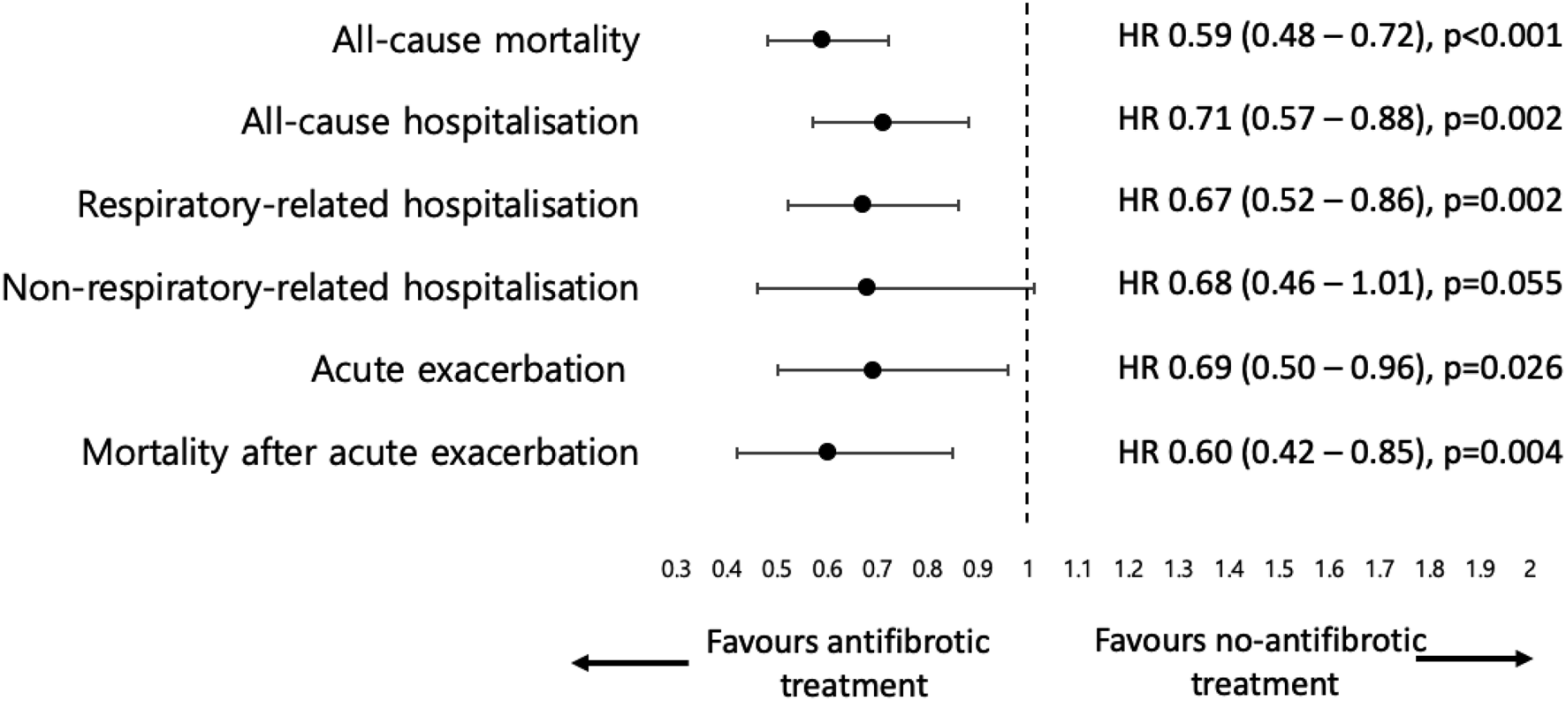
Forest plot demonstrating the risk of clinical outcomes in the antifibrotic and no-antifibrotic groups. Hazard ratios were calculated from univariable Cox proportional hazard analyses. *HR* hazard ratio.

In the univariate Cox analysis, antifibrotic treatment was associated with a significantly reduced risks of mortality [hazard ratio (HR), 0.59; 95% CI, 0.48–0.72; p < 0.001], all-cause hospitalisation (HR 0.71; 95% CI 0.57–0.88; p = 0.002), respiratory-related hospitalisation (HR 0.67; 95% CI 0.52–0.86; p = 0.002), AE (HR 0.69; 95% CI 0.50–0.96; p = 0.026), and mortality following AE (HR 0.60; 95% CI 0.42–0.85; p = 0.004) in IPF patients (Fig. 4). The risk of non-respiratory-related hospitalisation was lower in the antifibrotic group, although this difference was not statistically significant (HR 0.68; 95% CI 0.46–1.01; p = 0.055).

### Impact of antifibrotic treatment in the study subjects that include sildenafil users

Additional analyses were performed in study subjects including those treated with sildenafil. Propensity score matching yielded 530 matched pairs and their baseline characteristics are described in Supplementary Table 3. Patients who received sildenafil comprised 11.1% and 11.5% of the antifibrotic and no-antifibrotic group, respectively. As compared with the main analysis, inclusion of patients treated with sildenafil did not change the trend of beneficial effects of antifibrotic treatment. All-cause mortality (HR 0.63; 95% CI 0.52–0.75; p < 0.001), all-cause hospitalisation (HR 0.78; 95% CI 0.64–0.95; p = 0.013), respiratory-related hospitalisation (HR 0.76; 95% CI 0.61–0.95; p = 0.015), and mortality after acute exacerbation (HR 0.67; 95% CI 0.49–0.91; p = 0.011) were significantly lower with antifibrotic treatment as shown in Supplementary Figure S1. The risk of acute exacerbation tended to be lower in patients with antifibrotic treatment than in those treated without antifibrotic agents although it was not statistically significant (HR 0.77; 95% CI 0.58–1.04; p = 0.084).

## Discussion

Our study results suggest that antifibrotic treatment reduces the risks of all-cause mortality, hospitalisation (all-cause and respiratory-related), AE, and mortality after AE in the real-world cohort with IPF. The risk of all-cause mortality was approximately 40% lower in patients treated with antifibrotic agents than in those not administered antifibrotic agents.

The mortality benefit of antifibrotic agents is thought to persist during follow-up according to the results of our study and previous studies^28,29^. In open-label extension studies in which patients who finished the phase 3 trials were included to examine the long-term effect and safety profiles of antifibrotic medications, the effect of both pirfenidone and nintedanib on slowing the annual rate of FVC decline persisted beyond the clinical trial periods^28,29^. Therefore, it seems acceptable to assume that antifibrotic treatment also improves long-term clinical outcomes such as the risks of mortality and hospitalisation, as shown in our study. Interestingly, a recent study which analysed the clinical effectiveness of antifibrotic agents using an insurance database in the US found that the mortality benefit of antifibrotic treatment was observed only during the first 2 years of treatment^30^. The authors suggested some plausible reasons for this finding, one of which was that lung fibrosis continues despite antifibrotic treatment and vascular remodelling diminishes drug deposition in the lung parenchyma. It is unclear why a difference exists between the US data and our study, but the persistent mortality benefit shown in our study seems reasonable given the results of previous clinical trials and pooled analyses^10, 12^.

Patients with IPF experience frequent unexpected respiratory-related hospitalisations^31^, and AE is the most common cause of acute deterioration requiring hospitalisation^6^. In our study, the proportion of patients who experienced AE was significantly lower in the antifibrotic group. Previous studies have shown conflicting results with regards to AE. In the two replicate randomised phase 3 trials evaluating the efficacy of nintedanib, one showed that nintedanib significantly increased the time to the first AE, whereas the other found that the drug did not significantly affect this outcome^8^. However, in the pooled analysis, the number of patients who developed AE was lower in the nintedanib group than in the placebo group, and the time to the first AE also significantly decreased with nintedanib treatment (HR 0.53; 95% CI 0.34–0.83; p = 0.0047)^12^. In a recent Japanese study, perioperative administration of prophylactic pirfenidone was shown to be effective in preventing postoperative AE in lung cancer patients^32,33^. These data suggest that antifibrotic treatment may prevent the development of AE and support the findings of our study.

Aside from the incidence of AE, we found that the incidence of mortality following AE was also significantly lower in the antifibrotic group. To date, a few studies have suggested that antifibrotic treatment may reduce the risk of mortality after AE^6,11,34,35^. In a previous pooled analysis that showed that pirfenidone reduced the risk of unexpected hospitalisation, pirfenidone treatment was also found to significantly reduce the risk of mortality after hospitalisation (HR 0.56; 95% CI 0.32–0.99; p = 0.047)^11^. Given that AE is the most common cause of hospitalisation in IPF patients^6^, this finding may suggest that pirfenidone reduces the risk of mortality after AE, although this was not specifically investigated in that study. In a small study involving 20 IPF patients admitted to an intensive care unit (ICU) due to severe AE, pirfenidone was associated with a survival benefit^34^; patients who were taking pirfenidone at the time of admission (n = 11) had a significantly longer median survival after AE (137 days vs. 16 days, p = 0.009) than the control group (n = 9) and tended to have a lower ICU mortality rate (27.3 vs. 77.8%, p = 0.070). With regards to nintedanib, in the post-hoc analysis of the INPULSIS trials (n = 69), the 30-, 90-, and 180-day mortality rates after AE were numerically lower in patients treated with nintedanib than in those who received placebo (30-day: 20.6% vs. 40.0%; 90-day: 29.4% vs. 42.9%; 180-day: 35.3% vs. 57.1%)^35^. Along with the results of our study, these findings suggest that antifibrotic treatment reduces the risk of not only AE but also mortality after AE. Further studies are required to confirm this promising effect of antifibrotic treatment.

It should be noted that this study has some limitations. First, this was a retrospective observational study performed at a single centre, and this may limit the generalisability of our results. However, the baseline characteristics of our patients were similar to those of the patients included in previous reports^28,36^. In addition, single centre data could be advantageous in that patient management is less variable than in multicentre cohorts. Second, the follow-up duration was shorter in the antifibrotic group. The index date was the date of first prescription of an antifibrotic agent in the antifibrotic group, whereas it was the date of diagnosis in the no-antifibrotic group. Therefore, the shorter follow-up duration in the antifibrotic group was inevitable. Nonetheless, the favourable effects of antifibrotic agents remained the same even after adjustment for the follow-up period using a Cox proportional hazard model. Third, patients who were treated with sildenafil were not included in this study. In a previous study in which combination treatment with nintedanib and sildenafil was compared with nintedanib alone, combination treatment tended to improve the patients’ health-related quality of life and slow the rate of lung function decline^37^. Although these benefits were not statistically significant, sildenafil may provide an additional effect to antifibrotic agents, especially in patients with right heart dysfunction^38^. To eliminate any possible influence of sildenafil, we therefore excluded patients who received sildenafil. Instead, we performed additional analyses including patients who received sildenafil and the results were in favour of antifibrotic treatment. All-cause mortality, all-cause hospitalisation, respiratory-related hospitalisation, and mortality after exacerbation were significantly lower in the antifibrotic group than in the no-antifibrotic group. Acute exacerbation also tended to be lower with antifibrotic treatment. Lastly, more patients treated with pirfenidone than nintedanib were included in this study due to the limited accessibility to nintedanib. In South Korea, unlike pirfenidone, nintedanib is not yet covered by National Health Insurance. Although no clinical trial has directly compared the effectiveness of pirfenidone and nintedanib, it is assumed that pirfenidone and nintedanib exert similar beneficial effects given that both agents were demonstrated to reduce the decline rate in FVC by approximately 50%^7–9^.

In conclusion, our results suggest that in addition to reducing the rate of lung function decline, antifibrotic treatment may also reduce the risks of all-cause mortality, hospitalisation, AE, and mortality after AE in the real-world cohort with IPF.

## Supplementary information

Supplementary Information.
