# Supplementary material for: Antifibrotic treatment improves clinical outcomes in patients with idiopathic pulmonary fibrosis: a propensity score matching analysis
Source: Sci Rep. 2020 Sep 24;10:15620. doi: 10.1038/s41598-020-72607-1 (PMC7515864; doi:10.1038/s41598-020-72607-1)
Supplement: Supplementary file 1 — Supplementary Information. [file 41598_2020_72607_MOESM1_ESM.docx]

**Supplementary Data**

**Antifibrotic treatment improves clinical outcomes in patients with idiopathic pulmonary fibrosis: a propensity-score matching analysis**

Jieun Kang^1,2^, Minkyu Han^3^, Jin Woo Song^1^

^1^Department of Pulmonary and Critical Care Medicine, Asan Medical Center, University of Ulsan College of Medicine, Seoul, Republic of Korea

^2^Division of Pulmonary and Critical Care Medicine, Department of Internal Medicine, Ilsan Paik Hospital, Inje University College of medicine, Ilsan, Republic of Korea

^3^Department of Clinical Epidemiology and Biostatistics, Asan Medical Center, Seoul, Republic of Korea

Supplementary Table S1. Comparison of baseline characteristics between patients included in the matched cohort and those not included

|  | Total | Patients included in the matched cohort | Patients not included in the matched cohort | p-value |
| --- | --- | --- | --- | --- |
| No. of patients | 1213 | 948 | 265 |  |
| Age, years | 66.4 ± 8.0 | 65.8 ± 8.0 | 68.7 ± 7.5 | <0.001 |
| Male | 995 (82.0) | 785 (82.8) | 210 (79.2) | 0.205 |
| BMI | 24.3 ± 3.1 | 24.6 ± 2.9 | 23.3 ± 3.5 | <0.001 |
| Smoking status |  |  |  | 0.408 |
| Current | 148 (12.2) | 118 (12.4) | 30 (11.3) |  |
| Former | 777 (64.1) | 613 (64.7) | 164 (61.9) |  |
| Never | 288 (23.7) | 217 (22.9) | 71 (26.8) |  |
| FVC | 68.6 ± 16.2 | 67.1 ± 15.2 | 74.1 ± 18.5 | <0.001 |
| DL_CO_ | 55.2 ± 17.7 | 53.3 ± 16.6 | 62.7 ± 19.6 | <0.001 |
| Charlson Comorbidity Index | 1.9 ± 1.1 | 1.8 ± 1.1 | 1.9 ± 1.0 | 0.474 |

Data are presented as mean ± standard deviation or number (%) unless otherwise indicated.

BMI. body mass index; DL_CO_, diffusing capacity of the lung for carbon monoxide; FVC, forced vital capacity; No., number.

Supplementary Table S2. Causes of the first respiratory-related hospitalisation in patients with idiopathic pulmonary fibrosis

|  | Total | Antifibrotic | No antifibrotic |
| --- | --- | --- | --- |
| Number of patients | 280 | 105 | 175 |
| Acute exacerbation | 123 (43.9) | 46 (43.8) | 77 (44.0) |
| Pneumonia | 95 (33.9) | 39 (37.1) | 56 (32.0) |
| Disease progression | 27 (9.6) | 7 (6.7) | 20 (11.4) |
| Pneumothorax | 23 (8.2) | 11 (10.5) | 12 (6.9) |
| Mycobacterial | 5 (1.8) | 0 (0.0) | 5 (2.9) |
| Pulmonary embolism | 4 (1.4) | 1 (1.0) | 3 (1.7) |
| Others^*^ | 3 (1.1) | 1 (1.0) | 2 (1.1) |

Data are presented as number (%).

^*^Others included acute exacerbation of asthma (antifibrotic group), diffuse alveolar haemorrhage, and radiation pneumonitis.

Supplementary Table S3. Comparison of baseline characteristics between the antifibrotic and no antifibrotic groups of patients including those who received sildenafil

|  | Unmatched groups | |  | Matched groups | |  |
| --- | --- | --- | --- | --- | --- | --- |
|  | Antifibrotic | No antifibrotic | p-value | Antifibrotic | No antifibrotic | p-value |
| No. of patients | 608 | 751 |  | 530 | 530 |  |
| Age, years | 65.7 ± 7.8 | 67.0 ± 8.1 | 0.002 | 65.7 ± 7.6 | 65.9 ± 8.1 | 0.699 |
| Male | 503 (82.7) | 602 (80.2) | 0.235 | 438 (82.6) | 433 (81.7) | 0.748 |
| BMI | 24.9 ± 3.0 | 23.8 ± 3.1 | <0.001 | 24.8 ± 2.8 | 24.5 ± 3.0 | 0.176 |
| Smoking status |  |  | 0.895 |  |  | 0.366 |
| Current | 73 (12.0) | 84 (11.2) |  | 63 (11.9) | 60 (11.3) |  |
| Former | 389 (64.0) | 485 (64.6) |  | 333 (62.8) | 354 (66.8) |  |
| Never | 146 (24.0) | 182 (24.2) |  | 134 (25.3) | 116 (21.9) |  |
| FVC | 65.2 ± 14.0 | 69.5 ± 18.1 | <0.001 | 67.1 ± 13.4 | 67.0 ± 16.7 | 0.927 |
| DL_CO_ | 51.0 ± 16.1 | 56.0 ± 20.0 | <0.001 | 53.3 ± 15.2 | 53.3 ± 18.0 | 0.963 |
| Charlson Comorbidity Index | 1.8 ± 1.0 | 1.9 ± 1.1 | 0.399 | 1.8 ± 1.0 | 1.8 ± 1.1 | 0.977 |
| Sildenafil treatment | 67 (11.0) | 79 (10.5) | 0.792 | 59 (11.1) | 61 (11.5) | 0.923 |

Data are presented as mean ± standard deviation or number (%) unless otherwise indicated.

BMI, body mass index; DL_CO_, diffusing capacity of the lung for carbon monoxide; FVC, forced vital capacity; No., number.

Supplementary Figure S1. Forest plot demonstrating the risk of clinical outcomes in the study subjects including those who received sildenafil.


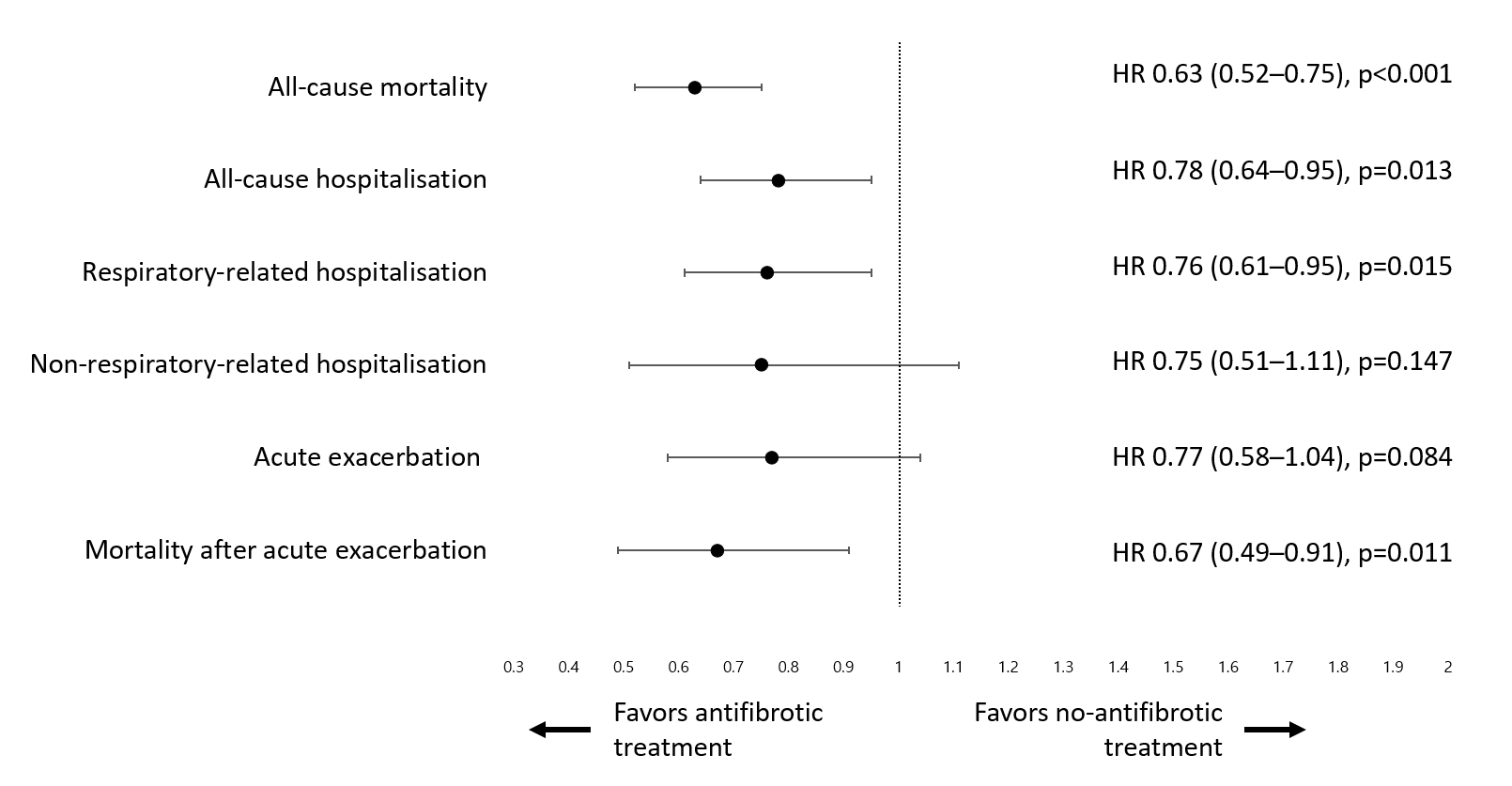


Hazard ratios were calculated from univariable Cox proportional hazard analyses. HR, hazard ratio.
